# Cervical and ocular vestibular evoked myogenic potentials in patients with Diabetic Peripheral Neuropathy

**DOI:** 10.1186/s13098-023-01068-z

**Published:** 2023-05-12

**Authors:** Jinying Zhang, Lichao Ye, Xuefeng Bai, Yali Huang, Jiayu Lin, Huapin Huang

**Affiliations:** 1 Department of Neurology, The Second Affiliated Hospital of Fujian Medical University, Quanzhou City, Fujian Province 362000 China; 2 Department of Endocrinology, The Second Affiliated Hospital of Fujian Medical University, No. 950 Donghai Street, Fengze District, Quanzhou City, Fujian Province 362000 China; 3 Department of Neurology, Fujian Medical University Union Hospital, Fuzhou City, Fujian Province 350000 China

**Keywords:** Diabetes, Diabetic peripheral neuropathy, Vestibular evoked myogenic potentials, Vestibular.

## Abstract

**Background:**

Diabetes causes impaired microarterial blood flow, demyelination and neuronal damage, which may lead to cochlear damage and vestibular malfunction. Vestibular evoked myogenic potentials (VEMP) is a simple, reproducible test. Cervical and ocular vestibular evoked myogenic potentials (cVEMP and oVEMP) can be explored in the saccadic-spinal and utriculo-ocular pathways in regular clinical practice.

**Objective:**

To evaluate possible vestibular evoked myogenic potential (VEMP) abnormalities in patients with diabetic peripheral neuropathy.

**Materials and methods:**

89 patients with Type 2 Diabetes in the present study consisted of three groups: 29 patients with no peripheral neuropathy (NDPN group), 26 patients with asymptomatic neuropathy (SDPN group), 34 patients with symptomatic neuropathy (DPN group). Meanwhile, 42 healthy subjects were recruited as controls. The clinical characteristics (including gender, age, body mass index (BMI), and illness duration), as well as lipids (including triglyceride (TG), cholesterol (TC), low-density lipoprotein (LDL), and high-density lipoprotein (HDL)), uric acid, fasting blood glucose (FBG), and glycated hemoglobin (HbA1c) were compared among the four groups. Four groups were assessed using two vestibular tests including oVEMP and cVEMP. Latency and amplitude parameters were analyzed from VEMP plots.

**Results:**

The latency of n10, p15 (oVEMP), p13, n23 (cVEMP) were significantly prolonged in the SDPN and DPN groups compared with the control and NDPN groups (p < 0.01), whereas latencies were similar in NDPN and the control groups. The amplitudes were not significantly different (p > 0.05). oVEMP latency p15 and cVEMP latency (p13, n23) were positively correlated with HbA1c, FBG, and illness duration, and oVEMP latency n10 was positively correlated with HbA1c and FBG. A nomogram, including FBG, HbA1C, HDL, TG, TC, LDL and group, was constructed to predict VEMP parameters and p13 was found to be independently associated with diabetic subgroups. Receiver operating characteristic curve (ROC) analysis showed good accuracy in predicting p13 in this nomogram. A user-friendly website has been created to facilitate the application of this prediction model ( https://fyey.shinyapps.io/VEMP_Model/ ).

**Conclusion:**

Patients with diabetic peripheral neuropathy may have vestibular dysfunction. VEMP may be useful in assessing vestibular impairment in diabetic patients.

## Introduction

Vestibular evoked myogenic potential (VEMP) is an objective method for assessing the otolithic apparatus and vestibular nerve conduction pathways in the vestibular system, such as the utricle and saccule, and is a myogenic potential recorded after stimulation of the otolithic apparatus by high-intensity acoustic signals [1]. Depending on the placement of the recording electrodes, VEMP examinations can be divided into cervical vestibular evoked myogenic potentials (cVEMPs) and ocular vestibular evoked myogenic potentials (oVEMPs). The oVEMP reflects the function of the superior vestibular pathway of the utricle and the cVEMP reflects the function of the inferior vestibular pathway of the saccule.

Diabetes mellitus is a metabolic disorder of multiple etiologies, characterized by chronic hyperglycemia and long-term complications [2]. In China, the proportion of people over 20 years of age with diabetes is reported to be 9.7% (about 92.4 million people), and the incidence of abnormal glucose tolerance is 15.5% (148.2 million people) [3]. The growing prevalence of diabetes and its complications has become a major health problem for Chinese nationals, placing a severe economic burden on society. Diabetes can cause a variety of clinical symptoms, among which balance dysfunction (dizziness, falls, etc.) is one of the symptoms commonly complained about by patients with this illness. Although the exact mechanism has not been sufficiently elucidated, Agrawal et al. [4] have indicated that the rate of vestibular dysfunction is 70% higher in diabetic patients than in healthy individuals. The risk of falls is markedly increased in DM and diabetic peripheral neuropathy (DPN), the role of DPN may promote vestibular impairments in these patients [5]. Meng Y et al. [6] performed vestibular function examination on 62 pre-diabetic patients and found that the incidence of vestibular impairment in diabetic patients was 71%. Vestibular impairment may be a major factor in the development of balance disorders in diabetics.

The detection of vestibular function in diabetics has received attention due to the increasing recognition of the impact of vestibular impairment on imbalance and falls in diabetics. Diabetic patients may develop vestibular dysfunction in the absence of significant vestibular symptoms [7]. Many researchers have identified various vestibular symptoms and vestibular test abnormalities in people with type 2 diabetes. While vestibular tests may be abnormal, these changes may be present in the absence of vestibular symptoms. In such cases, vestibular lesions can be identified by objective vestibular diagnostic testing tools, despite the absence of vestibular symptoms in patients with DM. Previous studies have used electronystagmography instruments to evaluate changes in vestibular function using spontaneous nystagmus tests, position tests, head shake tests, neck turn tests, temperature tests, and visual oculomotor system examinations (gaze test, sweep test, smooth tracking test) [8], but there are limitations due to the examination technique and patient cooperation. At present, VEMP testing constitutes an important part of the vestibular test battery and provides either diagnostic or assistive contributions in the clinical evaluation of common vestibular diseases such as Meniere’s disease (MD), vestibular neuritis (VN) and superior canal dehiscence syndrome (SCDS) [9]. In 2001, Perez et al. [6] assessed vestibular evoked potentials in experimentally induced type 2 diabetic animals, showing vestibular damage in the inner ear. However, the research literature on VEMP response in diabetics remains limited. The present study aimed to compare cVEMP and oVEMP in diabetes, subclinical diabetic peripheral neuropathy, DPN and healthy controls, and to assess the lesions of peripheral vestibular end organs by changes in parameters such as wave latency and amplitude, and to illustrate the impact of DPN on vestibular function in patients with DM.

### Materials and methods

This study was conducted at the Second Hospital of Fujian Medical University (Fujian, China), in accordance with the guidelines of the Declaration of Helsinki, and was approved by the Ethics Committee of the Second Hospital of Fujian Medical University [Ethics Approval No.: Lun Audit (Research) No. 213 of 2021].

### Participants

89 patients with type 2 diabetes who visited the Second Hospital of Fujian Medical University from April 2021 to November 2021 were enrolled in this study, including 57 males and 32 females with a mean age of (53.0 ± 11.9 years). There were 42 cases in the healthy control group, including 23 males and 19 females, with a mean age of (52.3 ± 5.6) years. (for details, see Table 1).The diagnosis of diabetes was based on the American Diabetes Association (ADA) (2021) diabetes diagnosis criteria [10]. The subjects had no existing/historical records of any otological disorders, nor any historical or existing vestibular complaints (such as dizziness, vertigo, nausea). Patients present with a history of head-and-neck injury, limited neck mobility, history of head and neck surgery, central nervous system disorders; those who present with diseases involving external or middle ear, those who present with a history of usage of drugs such as ototoxic drugs, antiepileptics, muscle relaxants; those who were unable to cooperate were excluded from the study. The patients gave permission for all of the procedures involving clinical tests and data collection, with approval by the ethical committee of the Second Hospital of Fujian Medical University, China. General information including gender, age, body mass index, duration of diabetes, glycated hemoglobin (HbA1c), fasting blood glucose(FBG), blood pressure, smoking history, triglycerides, total cholesterol, low-density lipoprotein cholesterol (LDL-C), and high-density lipoprotein cholesterol (HDL-C) were recorded for all participants.

### Test used

All subjects were examined using an electromyographic evocator (Keypoint 9033A07, Focus), including nerve conduction velocity measurement, cVEMP test, and oVEMP test. All subjects were tested in a quiet environment.

### Nerve conduction velocity examination

All enrolled patients were examined by a neurologist specializing in nerve conduction velocity. Diabetic peripheral neuropathy was diagnosed using the diagnostic criteria of the Expert consensus on the diagnosis and management of diabetic neuropathy (2021 Edition) [11]. Diagnosis of distal symmetric polyneuropathy or multiple peripheral neuropathies by nerve conduction velocity examination. 89 patients with Type 2 Diabetes in the present study consisted of three groups: 29 patients with no peripheral neuropathy (NDPN group), 26 patients with asymptomatic neuropathy (SDPN group), 34 patients with symptomatic neuropathy (DPN group).

The diagnostic criteria for diabetic peripheral neuropathy include the following three points: (1) definite presence of diabetes; (2) presence of clinical and/or electrophysiological evidence of peripheral neuropathy; and (3) exclusion of other causes of peripheral neuropathy [11]. The clinical manifestations are symmetrical pain and paresthesia, with paresthesia in the early stage and sensory loss in the late stage. The symptoms of lower limbs are more common than those of upper limbs. Electromyography can confirm peripheral neuropathy and assist in determining its type as well as its severity. In asymptomatic diabetic patients, electrophysiological examination can help detect subclinical peripheral neuropathy.

### VEMP test

VEMP test was executed using a Keypoint (9033A07, Focus) with the following settings and equipment: 2 channels; The velocity is 10 ms/D; sensitivity 0.2 mV/D; Filters range from 5 Hz to 5 kHz; click sound (300–500 stimuli for 0.1 ms at 5 Hz each); single-channel calibrated stereo headphones and surface electrodes. Consecutive runs were performed to confirm the reproducibility of biphasic waveform for cVEMP and oVEMP. Conversely, cVEMP and oVEMP responses were absent when reproducibility was lacking. The presence of a VEMP reaction was evaluated, and the parameters included latencies (ms) and amplitudes (mV). (1) cVEMP test: The patient was placed in the supine position. Two active electrodes were placed on the upper two thirds of the bilateral sternocleidomastoid (SCM) muscles with the reference electrode on the sternoclavicular joint. Subjects were instructed to lift the head from the pillow so that the sternocleidomastoid muscle contracted and maintained a muscle tension greater than 50 uV. Thereafter, the examiner adjusted the headset stimulus to 135 dB SPL for an average of approximately 300–500 repetitions until a steady waveform was obtained and labeled p13, n23 ^[[12][13]]^. The presence of cVEMP was confirmed when reproducible short-latency biphasic waveforms appeared at certain latencies. The latencies of p13 and n23 were measured. Typical cVEMP waveforms were bidirectional, with the positive wave latency near 13 ms, labeled p13, and the negative wave appearing near 23 ms, labeled n23. The recorded EMG activity was corrected, which reflects the reflected potential originating from the saccule. (2) oVEMP test: The recording electrode was located just below the lower lid margin of each eye in line with the pupil. Subjects were asked to gaze maximally upward by looking at the target point located 60–70 cm from the eyes with a visual angle of 25°, and oVEMPs were recorded from the contralateral extraocular muscles [14]. The n10-p15 composite waveform was recorded as negative followed by positive, and the trough with an upward trend is marked as n10 about 10 ms after the sound is given, and the peak with a downward trend after n10 is marked as p15. The recorded waveform with good reproducibility after 3 repeated utterances of the same intensity was considered as the oVEMP response waveform [12].

## Data analysis

The data was analyzed using SPSS software(Version 26) and R (version 4.2.2). The quantitative results were presented with the mean ± standard deviation. Data between groups were analyzed by one-way ANOVA, and multiple comparisons were executed by LSD method. The elicited rates of oVEMP and cVEMP testing were compared in two groups by chi-square test. Correlation coefficient and prediction capability were assessed by Pearson correlation analysis and ROC analysis, respectively. The “ggplot2” and “Regplot” R packages were used to draw figures. DynNom: Visualising statistical models using dynamic nomograms. A *p*-value < 0.05 was considered to indicate statistical significance.

## Results

### Comparison of baseline information of the four groups

The demographic features and hematological examination of the groups are shown in Table 1. There was no significant difference between the groups in terms of gender, age, BMI, lipids (P > 0.05). There was no significant difference in underlying comorbities rates (Hypertension, CVD and CAD) of NDPN, SDPN and DPN groups (P > 0.05). There were significantly higher fasting blood glucose and HbA1c levels in the NDPN, SDPN, and DPN groups compared to the control group (P < 0.01). HbA1c, fasting blood glucose, and illness duration were larger in the DPN group than in the NDPN and SDPN groups, but there was no significant difference between the NDPN group and the SDPN group.

**Table 1:** Comparison of baseline information of the four groups

	Control group	NDPN group	SDPN group	DPN group
n (M/F)	42(23/19)	29(19/10)	26(15/11)	34(23/11)
Age (/year)	52.3 ± 5.6	52.9 ± 7.6	51.3 ± 12.8	51.6 ± 13.1
BMI(kg/m2)	22.5 ± 2.1	23.5 ± 2.6	23.6 ± 2.3	23.1 ± 3.1
TG(mmol/l)	1.7 ± 1.1	1.9 ± 1.3	1.8 ± 0.8	1.8 ± 1.5
HDL-C(mmol/l)	1.3 ± 0.4	1.1 ± 0.3	1.2 ± 0.1	1.3 ± 0.5
LDL-C(mmol/l)	2.9 ± 1.1	3.0 ± 1.2	3.2 ± 1.0	3.4 ± 1.1
TC(mmol/l)	4.6 ± 1.4	4.7 ± 1.2	4.7 ± 1.0	5.2 ± 1.4
UA(umol/L)	300.1 ± 88.2	355.7 ± 106.5	308.9 ± 64.4	325.3 ± 89.7
HbA1c(%)	5.6 ± 0.4	8.6 ± 2.1^#^	8.7 ± 2.1^#^	10.9 ± 2.5^#**^
FBG(mmol/L)	5.1 ± 0.5	8.7 ± 3.2^#^	8.9 ± 3.0^#^	10.9 ± 4.9^#*^
Illness duration(/year)	-	4.2 ± 4.5^#^	4.5 ± 5.1^#^	7.3 ± 5.5^#*^
Hypertension (%)	-	14 (48.27)	13 (50.00)	18 (52.94)
CAD (%)	-	3 (10.34)	3 (11.54)	4 (11.76)
CVD (%)	-	2 (6.89)	2 (7.69)	3 (8.82)

F = Female, M = Male; BMI = body mass index; TG = Triglyceride; HDL-C = high-density lipoprotein; LDL-C = Low density lipoprotein; TC = Cholesterol; UA = Uric acid; HbA1c = glycated hemoglobin; FBG = fasting blood glucose; CAD = coronary artery disease; CVD = cerebrovascular disease;

Compared to healthy control and NDPN groups, ^#^p < 0.01; Compared to SDPN group, ^*^p < 0.05, ^**^p < 0.01

### Comparison of VEMP elicitation rates in subjects

A total of 84 ears were used in healthy controls, of which 80 ears elicited oVEMP (elicitation rate of 95.2%) and 82 ears elicited cVEMP (elicitation rate of 97.6%). Diabetic subjects had a total of 178 ears, of which 160 ears elicited oVEMP (elicitation rate of 90.1%) and 163 ears elicited cVEMP (elicitation rate of 91.6%). There was no significant difference in oVEMP and cVEMP elicitation rates in healthy controls compared with diabetic subjects (oVEMP: χ^2^ = 1.54; cVEMP: χ^2^ = 2.70, P > 0.05).

3.3 Comparison of latencies and amplitudes in oVEMP and cVEMP tests between the four groups.

The results from the oVEMP and cVEMP tests are shown in Table 2. When the oVEMP parameters of the groups were analysed, the subjects in the SDPN and DPN groups had longer n10 and p15 latencies than those in the NDPN and control groups (P < 0.01). For cVEMP, the SDPN and DPN groups had longer p13 and n23 latencies than the control and NDPN groups (P < 0.01). There was no significant difference in latencies (p13, n23) of SDPN group and DPN group (P > 0.05). No significant differences existed between the group amplitudes.

**Table 2:** oVEMP and cVEMP parameters of the control, NDPN, SDPN and DPN groups

	Control group	NDPN group	SDPN group	DPN group
Ln10(ms)	11.3 ± 0.9	10.9 ± 0.9	12.2 ± 1.1^#^	12.5 ± 1.0^#^
Lp15(ms)	14.9 ± 0.8	15.1 ± 1.4	17.0 ± 1.0^#^	16.8 ± 1.2^#^
LoAmp(uV)	1.8 ± 1.0	1.6 ± 0.7	1.8 ± 1.2	1.8 ± 1.5
Rn10(ms)	11.1 ± 0.8	11.0 ± 1.1	12.2 ± 1.3^#^	12.5 ± 1.0^#^
Rp15(ms)	15.1 ± 1.0	15.1 ± 1.3	16.7 ± 1.5^#^	16.7 ± 1.2^#^
RoAmp(uV)	1.6 ± 1.2	1.7 ± 1.1	1.6 ± 1.1	1.5 ± 1.0
Lp13(ms)	12.1 ± 0.9	12.3 ± 0.8	13.3 ± 1.5^#^	13.6 ± 1.6^#^
Ln23(ms)	21.8 ± 1.1	21.8 ± 1.1	23.6 ± 1.9^#^	24.1 ± 1.7^#^
LcAmp(uV)	114.1 ± 58.5	94.6 ± 51.3	90.9 ± 62.3	99.8 ± 46.4
Rp13(ms)	12.1 ± 0.8	12.4 ± 0.8	13.3 ± 1.5^#^	13.7 ± 1.5^#^
Rn23(ms)	21.8 ± 1.4	21.8 ± 1.7	23.7 ± 2.1^#^	24.2 ± 1.6^#^
RcAmp(uV)	114.6 ± 58.0	82.2 ± 47.0	89.3 ± 42.1	99.0 ± 45.0

L = Left, R = Right; n10 = n10 latency, p15 = p15 latency, p13 = p13 latency, n23 = n23 latency; o = ocular vestibular evoked myogenic potential; c = cervical vestibular evoked myogenic potential; Amp = Amplitude.

NDPN refers to diabetes without neuropathy. SDPN refers to diabetes mellitus combined with asymptomatic neuropathy. DPN refers to diabetes mellitus combined with symptomatic neuropathy compared to healthy control and NDPN groups, #p < 0.01.

### Correlations between HbA1c, FBG, illness duration, and oVEMP and cVEMP parameters

The correlation coefficients between HbA1c, FBG, duration of illness and oVEMP and cVEMP parameters are shown in Table 3. A statistically significant positive correlation between the latencies (oVEMP p15 and cVEMP p13, n23) and HbA1c, FBG, and illness duration was observed. The values of oVEMP p15 and cVEMP p13, n23 also increased with HbA1c, FBG, and duration of illness.

**Table 3:** The correlation coefficients of HbA1c, FBG, and illness duration with VEMP parameters

	HbA1c	FBG	illness duration
Ln10	0.279^**^	0.311^**^	0.125
Lp15	0.492^**^	0.456^**^	0.231^**^
LoAmp	0.102	0.048	0.009
Rn10	0.365^**^	0.370^**^	0.244^**^
Rp15	0.358^**^	0.369^**^	0.242^**^
RoAmp	0.006	0.020	0.071
Lp13	0.201^*^	0.256^**^	0.224^*^
Ln23	0.247^**^	0.227^*^	0.381^**^
LcAmp	-0.079	-0.112	-0.031
Rp13	0.242^**^	0.267^**^	0.245^**^
Rn23	0.269^**^	0.288^**^	0.326^**^
RcAmp	-0.083	-0.167	-0.069

L = Left, R = Right; n10 = n10 latency, p15 = p15 latency, p13 = p13 latency, n23 = n23 latency; o = ocular vestibular evoked myogenic potential; c = cervical vestibular evoked myogenic potential; Amp = Amplitude.

HbA1c = glycated hemoglobin; FBG = fasting blood glucose

* P < 0.05

** P < 0.01

### Comparison of the VEMP parameters between the left and right side aliases

There is no significant difference between the latencies and amplitudes of the left and right sides (p > 0.05, Table 4). It is indicated that the differences between subjects at the left and right side do not affect the interpretation of VEMPs results. Since no difference was found between the latencies and amplitudes from the left and right ears, the mean latencies and amplitudes recorded from both ears were evaluated in the following study.

**Table 4:** Comparison of VEMP parameters between the left and right sides

side	n	oVEMP			cVEMP		
		n10	p15	Amp	p13	n23	Amp
left	131	11.70 ± 1.16	15.68 ± 1.50	1.67 ± 1.12	12.74 ± 1.33	22.70 ± 1.78	101.49 ± 55.01
right	131	11.67 ± 1.21	15.66 ± 1.41	1.58 ± 1.11	12.81 ± 1.34	22.76 ± 1.97	98.35 ± 50.51
t-value		0.370	0.212	1.845	−1.360	−0.832	0.843
P-value		0.712	0.832	0.067	0.176	0.407	0.401

### Relationship of VEMP parameters with multiple clinical characteristics

**Fig. 1:**
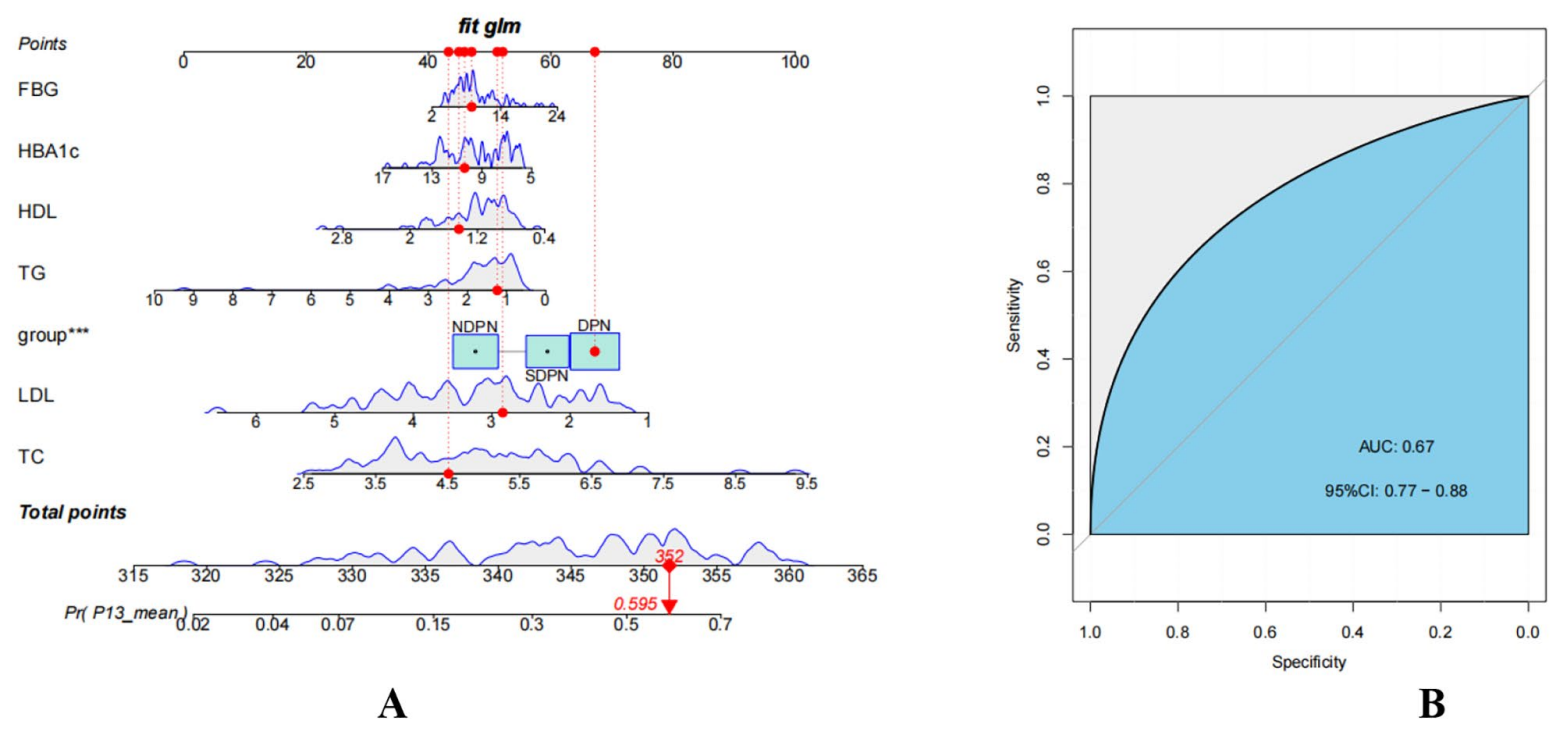
Establishment of nomograms for predicting p13 in diabetic patients (A) Nomogram for predicting p13 in diabetic patients using group, FBG, HbA1C, HDL, TG, TC, LDL. (B) ROC curves showing the accuracy of predicting p13 on the basis of nomograms ***P < 0.01

We used a Cox regression model to determine the factors that affect the VEMP parameters of patients with Type 2 Diabetes to construct a nomogram. FBG, HbA1C, HDL, TG, TC, LDL, and group were included in the nomogram. The nomogram is displayed in Fig. 1. When we used the nomogram, we first needed to determine its position on different variable axes, find the corresponding points on the top axis, add the point values of all variables together, and draw a vertical line down based on this sum point to predict VEMP parameters (Fig. 1A), and found that p13 was independently associated with diabetic subgroups. ROC analysis showed that the nomogram had good accuracy in predicting p13 (Fig. 1B). In addition, a user-friendly website has been created to facilitate the application of this prediction model ( https://fyey.shinyapps.io/VEMP_Model/ ). If you press Predict, the Graphical Summary will indicate ERROR: An error has occurred. Check your logs or contact the app author for clarification”, then press “Quit”.

## Discussion

In this study, vestibular function was assessed in patients with type 2 diabetes. The results indicated that the latency (n10, p15, p13, n23) was prolonged in patients with the SDPN group and DPN group, compared to the NDPN group and control group. The latency was correlated with FBG, HbA1c, and duration of illness. VEMP abnormalities are present in patients with diabetic peripheral neuropathy.

The maintenance of the body’s balance posture involves the combined action of the vestibular organs, visual senses, and proprioception [15]. The patient’s ability to retain balance is successively diminished when vestibular function is impaired. Diabetes mellitus may enhance the risk of vestibular impairment [16]. Perez et al. [17] confirmed functional impairment of the vestibular portion of the inner ear in the metabolic state of diabetes using vestibular end-organ testing and a diet-induced type 2 diabetes animal model. The research concluded that microangiopathy is the major cause of abnormal vestibular function [18] and may be related to the cochlear ganglion having a common nerve sheath adjoining to the vestibular ganglion. Microangiopathy leads to ischemia of the vestibular structures and alters fluid metabolism in the inner ear, causing symptoms of vestibular dysfunction such as tinnitus, hearing loss, and vertigo in diabetic patients [19–22]. Pathogenesis may involve degenerative changes due to sensory or motor polyneuropathy, a common complication of type 2 diabetes. Alterations in polyol pathways, succinate accumulation [23], late glycosylation end products, and hemodynamic factors leading to myelin loss and axonal degeneration are possible pathogenic mechanisms [24]. Gawron et al. [25] suggested that diabetic vestibular impairment is mainly related to diabetic peripheral neuropathy and microangiopathy. The vestibular receptor end organ is supplied only by the vagus artery, which is microvascular, and diabetic microangiopathy causes peripheral damage to the vestibular system when the vagus artery is under-supplied or occluded. Agrawal et al. [26] compared the vestibular function in patients with diabetes mellitus combined with peripheral neuropathy and diabetes mellitus without any peripheral damage. The results showed a higher incidence of vestibular dysfunction in those with combined diabetes mellitus and peripheral neuropathy, suggesting that the pathogenesis of diabetic vestibular impairment may be related to diabetic peripheral neuropathy and microangiopathy.

The detection of diabetic vestibular function has received attention as the impact of vestibular impairment on balance and falls in diabetic patients has been progressively recognized. Different tests, such as Pure Tone Audiometry, Video Head Impulse Test, VEMP, are used to assess vestibular function. The VEMP is a short-latency evoked potential that allows non-invasive assessment of the vestibular-collic and vestibular-ocular pathways. It is divided into cVEMP and oVEMP. The cVEMP assesses the saccule, inferior vestibular nerve, and sternocleidomastoid muscle. oVEMP assesses the utricle and superior vestibular nerve function [29]. VEMP evaluates clinically common vestibular disorders such as superior canal dehiscence syndrome (SCDS), Ménière’s disease (MD), and vestibular neuritis (VN) [30].

Recently, the function of the vestibular system in diabetics has been investigated using VEMP. Data on VEMP in diabetics is still limited. In a previous report by Kalkan et al. [27], latencies were examined with cVEMP in non-insulin-dependent diabetic patients with or without polyneuropathy, and no prolonged latencies were found in either group. These outcomes are in conflict with the results of our study, presumably due to their small latency range. The disagreement data on VEMP reactions (including ours) may be due to the comparatively few studies in the literature focusing on the vestibular system and possibly the relatively few participants. Alternatively, it may be related to clinical factors that do not match between study groups, such as other diabetes-related complications or the presence of vestibular symptoms of varying degrees. Similar to our study, Kamali et al. [28] evaluated 14 diabetic patients with peripheral neuropathy, 10 diabetic patients without peripheral neuropathy and 24 healthy controls. Statistically significant differences in p13 and n23 latencies existed between the groups, with diabetics showing prolonged cVEMP latencies, but the amplitude of cVEMP in did not vary between the groups. The prolonged latency may be due to post-vagal demyelinating lesions or polyneuropathy, which is common in type 2 diabetes. In the present study, the latencies of n10 and p15 for oVEMP and p13 and n23 for cVEMP were longer in the SDPN and DPN groups, compared to the NDPN group and control group, which is a significant difference. Additionally, the nomogram, including group, FBG, HbA1C, HDL, TG, TC, LDL, was established to create an applicable clinical evaluation instrument to predict VEMP parameters among diabetic patients and ROC analysis revealed the excellent accuracy of the nomogram in predicting p13(AUC 0.67, 95% CI 0.77–0.88). Furthermore, it is noteworthy that diabetic grouping is a relevant factor affecting p13 latency, suggesting that the pathogenesis of diabetic vestibular injury may be related to diabetic peripheral neuropathy.

VEMP appears to be more significant in the detection of subclinical lesions. In the present study, subjects with vestibular symptoms were excluded to investigate vestibular involvement in patients with diabetic peripheral neuropathy without significant vestibular symptoms. Rigon et al. [31] reported that the vestibular system may be impaired in diabetic patients without vestibular symptoms. Konukseven et al. [7] introduced a new term called “subclinical vestibular neuropathy” to describe diabetic patients with vestibular involvement but without vestibular symptoms. Similar to these reports, despite the absence of vestibular symptoms, the VEMP latency was prolonged in the SDPN and DPN groups compared to the control and NDPN groups in the present study. These findings may support the presence of vestibular involvement in patients with diabetic peripheral neuropathy without significant vestibular complaints. This result suggests that vestibular detection is affected in patients with peripheral neuropathy in type 2 diabetes, despite the absence of significant vestibular symptoms.

Current studies on the effects of diabetes duration and glycemic control on vestibular function are inconsistent. Agrawal et al. [4] concluded that longer duration of diabetes or higher glycosylated hemoglobin levels resulted in a higher incidence of vestibular dysfunction. Ward et al. [32] concluded that duration of diabetes and glycemic control were not significantly correlated with vestibular impairment. In our study, we found that cVEMP and oVEMP latencies were correlated positively with the duration of diabetes, FBG and HbA1c. The latencies were prolonged with increased glycosylated hemoglobin and fasting glucose levels, indicating that poor glycemic control may exacerbates vestibular impairment. Chronic hyperglycemia, insulin resistance [33] and abnormal lipid metabolism are various risk factors that continue to accumulate, aggravating microangiopathy and promoting atherosclerosis, leading to vestibular damage. Hyperglycemic states can cause damage to the vestibule through a variety of pathways including oxidative stress, hypoxia and local ischemia, activation of polyol pathways, and increased concentrations of glycosylation end products. The severity of diabetes is closely associated with vestibular dysfunction, suggesting that this complication may be an index of fall risk in diabetic patients. The sample size of this research was comparatively small, and further expansion of the sample size is required for validation to determine the temporal relationship between the development of clinical and objective vestibular and/or auditory manifestations.

## Conclusion

Diabetic microvascular complications have so far been defined as retinopathy, nephropathy and neuropathy. Polyneuropathy and retinopathy in diabetes mellitus lead to an increased risk of falls due to gait unsteadiness among many patients. However, the risk of falls and dizziness in DM patients should also take into account the contribution of damage to the vestibular end organs. Vestibular function impairment as an important complication of diabetes mellitus, leading to balance dysfunction in diabetic patients and severely affecting the quality of life. The results of oVEMP and cVEMP assays may be affected by metabolic factors. By studying the characteristics of vestibular function changes in diabetics, it is beneficial to increase the understanding of vestibular impairment in diabetics, provide a basis for early detection and rehabilitation of vestibular impairment in diabetics, reduce the incidence of falls in diabetics, and improve quality of life.

## Data Availability

The datasets used or analysed during the current study are available from the corresponding author on reasonable request.
